# Supplementary material for: Identification of a guanine-specific pocket in the protein N of SARS-CoV-2
Source: Commun Biol. 2022 Jul 16;5:711. doi: 10.1038/s42003-022-03647-8 (PMC9288159; doi:10.1038/s42003-022-03647-8)
Supplement: Supplementary file 2 — Supplementary Information [file 42003_2022_3647_MOESM2_ESM.pdf]

Supplementary Figure 1

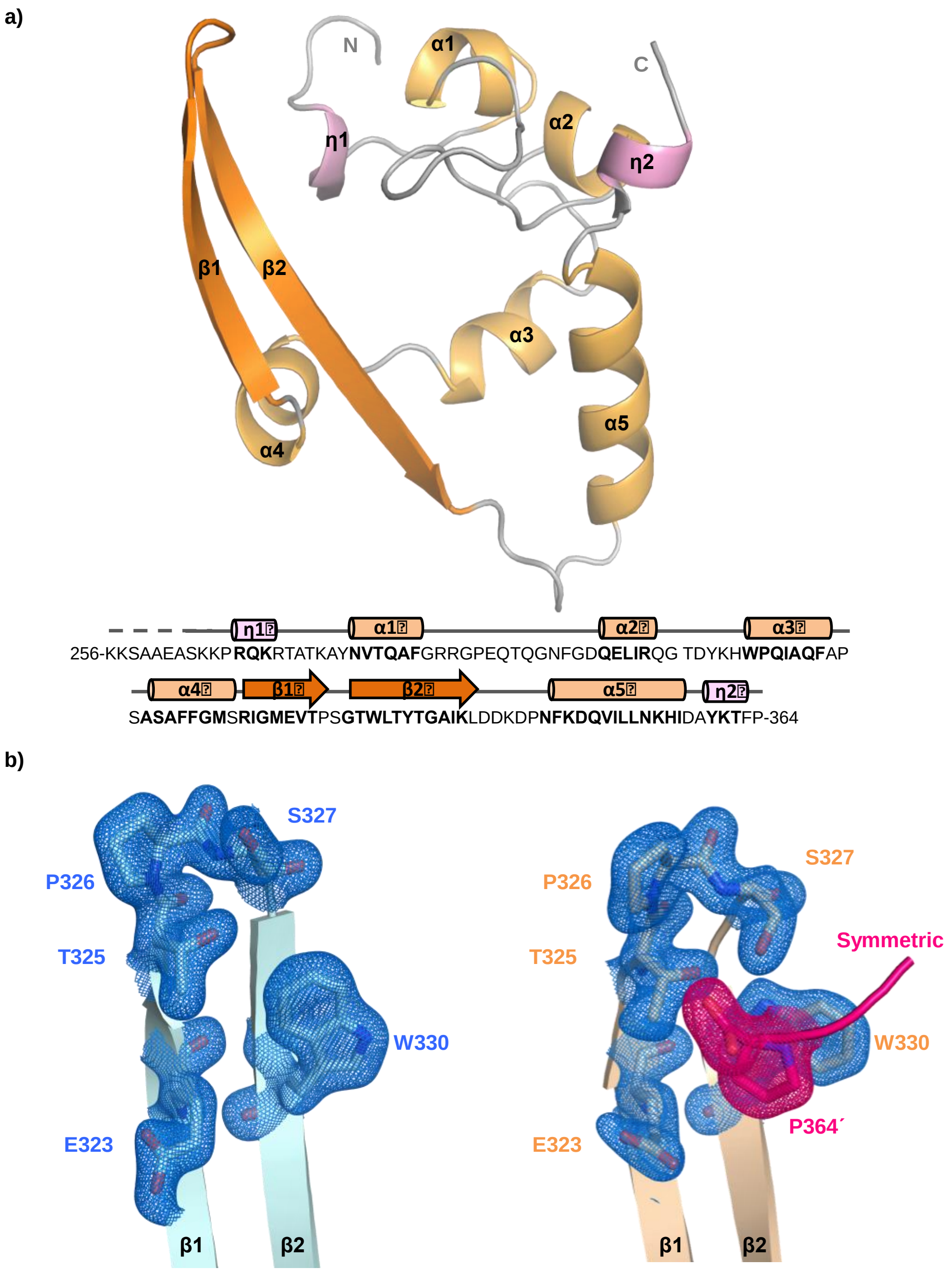

Supplementary Fig. 1. Structure of SARS-CoV-2 N^{CTD} and conformations of the β-hairpin.
a) Cartoon representation of N^{CTD} with secondary structural element colored in grey (loops), light orange (α-helices), dark orange (β-strands), and pink (3_{10} helices). The sequence of N^{CTD} is shown below the structure. **b**) Close view of the β-hairpin in the open (left; blue) and closed (right; orange) conformations. The side chains of the residues that rotate within the different confirmation are shown in sticks. The electron density map 2Fo-Fc (σ=1) is represented in blue. The symmetric molecule is coloured in magenta and the C-terminal Pro364 is shown in sticks and its electron map in magenta. Nitrogen and oxygen atoms are colored in blue and red, respectively. Secondary structural elements and residues are numbered and labeled in order from N to C terminus.

Supplementary Figure 2

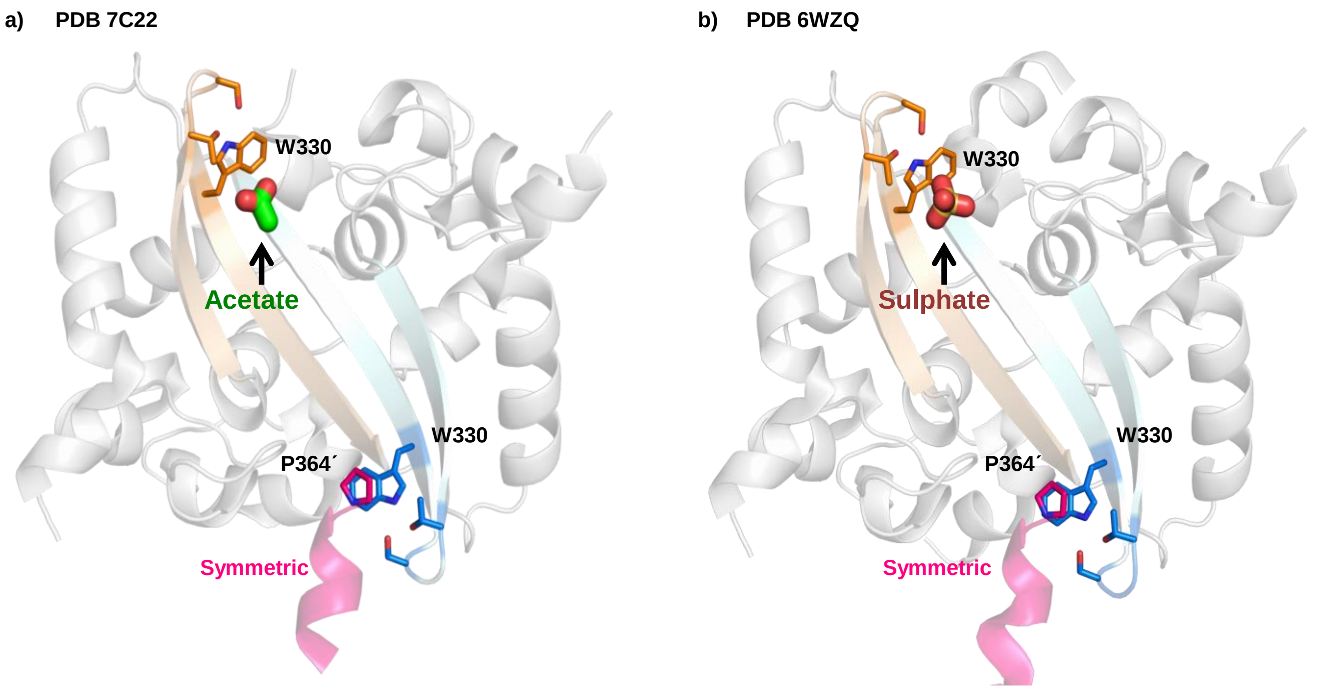

Supplementary Figure 2. **Acetate and Sulphate molecules bound to N^{CTD} W330.**
Cartoon representation of SARS-CoV-2 N^{CTD} structures PDB 7C22 (**a**) and PDB 6WZQ (**b**). The β-hairpin of each monomer is colored in pale orange and pale cyan. The helical core is coloured in white and the symmetric in magenta. Residues of the β-hairpin and the symmetric Pro364´ are shown in sticks with carbon atoms in the same colour to the subunit to which they belong. The acetate molecule is shown in sticks with carbon atom in green. The sulphate molecule is shown in sticks with sulphur atom in yellow and oxygen atoms in red.

Supplementary Figure 3

a)

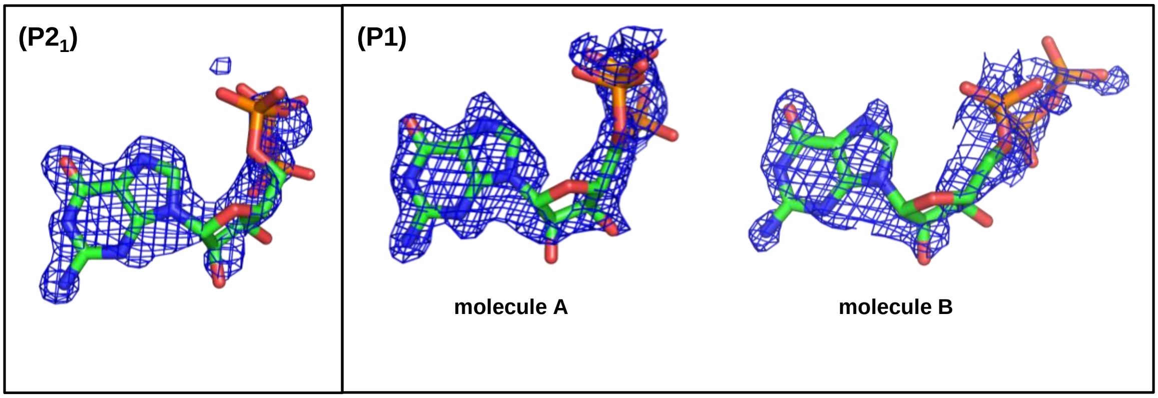

b)

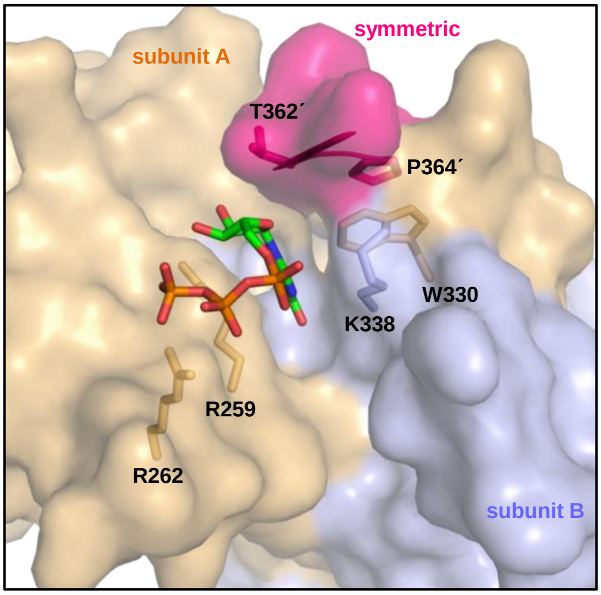

Supplementary Figure 3. (a) Simulated anneal omit maps of the GTP molecules. Maps are represented in blue color at σ=1, carve 1.6. **(b)** Surface representation of the GTP binding pocket. Each subunit of the dimer is colored in blue and orange, respectively, and the symmetric molecule in black. The side chains of the W330 and neighbouring residues are represented in sticks in the same colour the subunit to which they belong. The GTP is shown in sticks with carbon, nitrogen, oxygen and phosphorus atoms coloured in green, blue, red and orange colours, respectively.

Supplementary Figure 4

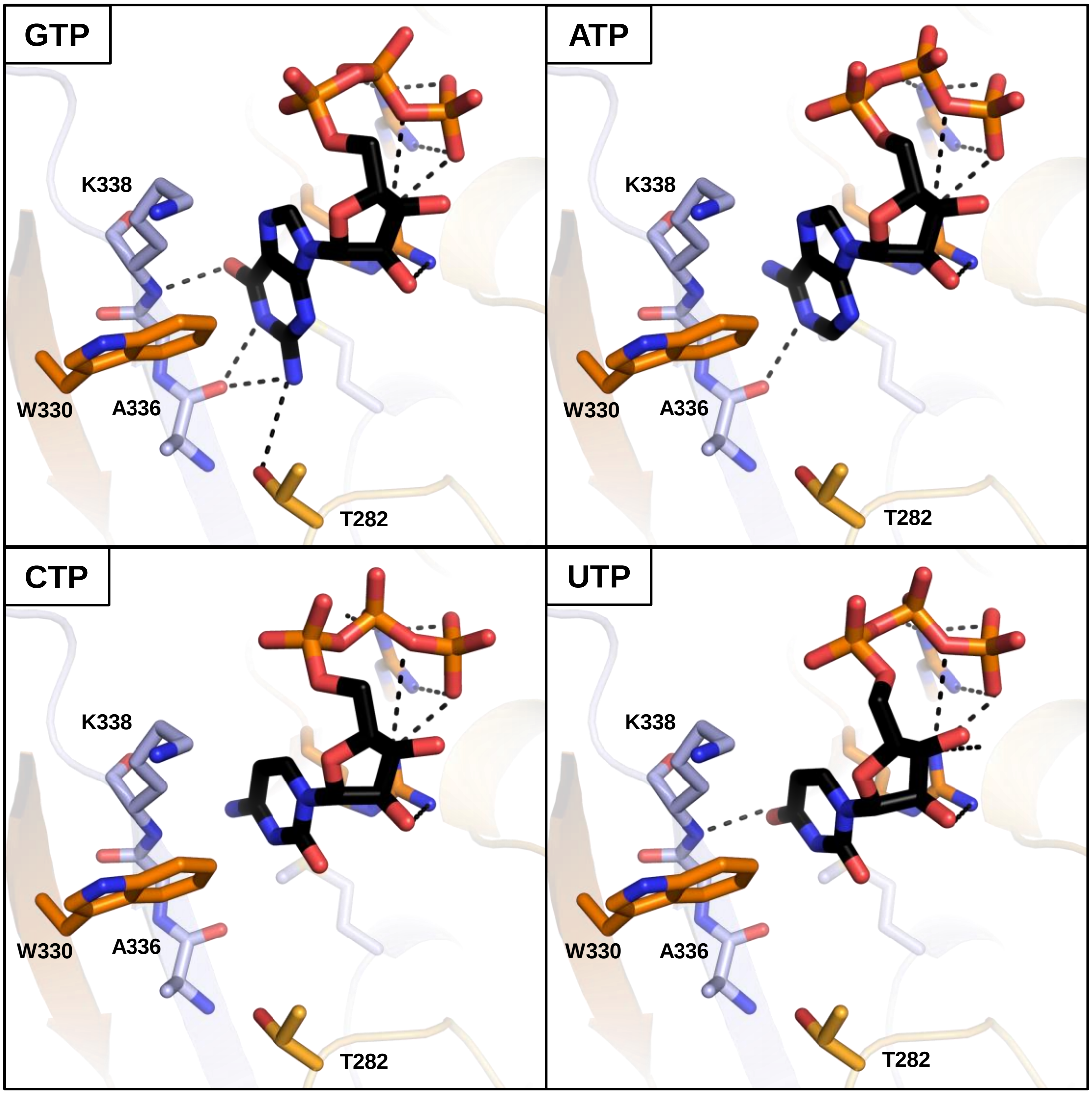

Supplementary Figure 4. Selectivity of the GTP binding site of N^{CTD} for guanine base. The ribonucleotides ATP, CTP and UTP are superposed over the GTP molecule. The side cain of the residues that mediate interaction with GTP are shown in sticks. Structural elements and carbon atoms of each monomer of the dimer are represented in orange and blue colors.Ligands are represented in sticks, with carbon in black. Nitrogen, oxygen and phosphorous atoms are colored in blue, red and orange, respectively.

Supplementary Figure 5

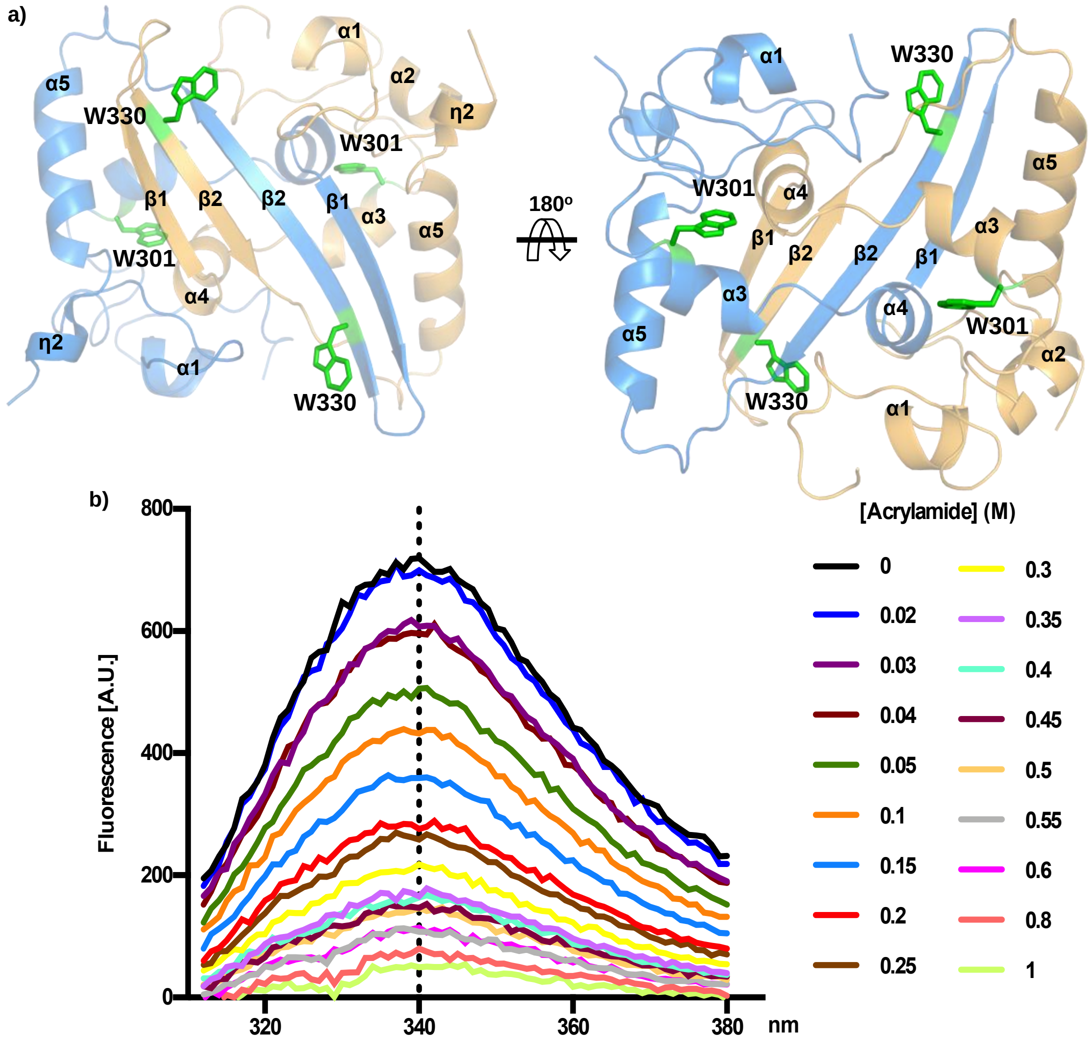

Supplementary Figure 5. **Tryptophan fluorescence quenching of SARS-CoV-2 N^{CTD}.**
a) Two rotated views of N^{CTD} dimer. Each monomer is colored in blue and orange. Tryptophan residues are shown in sticks, labelled, and coloured in green. Secondary structural elements numbered and labeled in order from N to C terminus. **b**) Tryptophan fluorescence emission of N^{CTD} from 312 to 380 nm in absence or increasing concentration of acrylamide.

Supplementary Figure 6

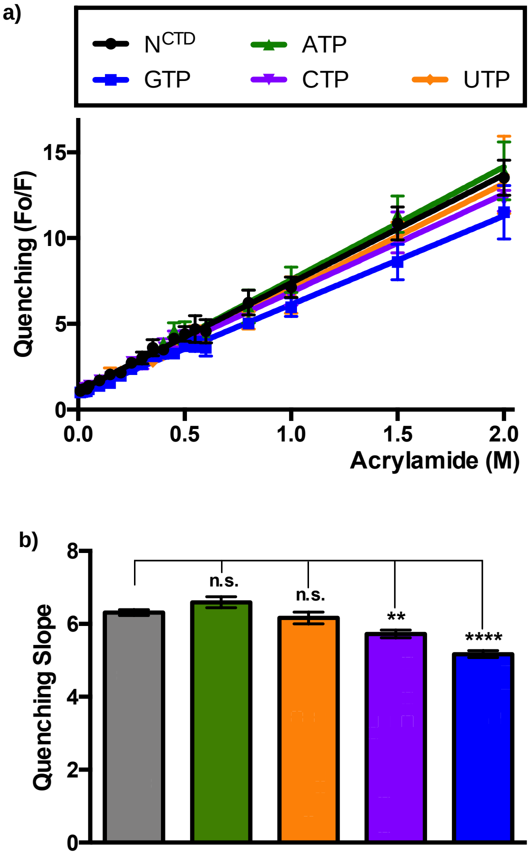

Supplementary Figure 6. **Interference of oxynucleotides in the quenching of the tryptophan fluorescence of SARS-CoV-2 N^{CTD}.**

a) Quenching of the tryptophan fluorescence emission at 340 nm by acrylamide in absence or presence of ATP, GTP, CTP, UTP. The quenching is represented by the ratio of fluorescence in absence of acrylamide (Fo) by the fluorescence in presence of acrylamide (F). **b)** Bar graph of the quenching slope and standard error in absence or presence of oxynucleotides. Statistical differences are indicated with asterisks above each bar (**** *p* <0.0001, ** *p* <0.01, n.s. *p* >0.05).

Supplementary Figure 7

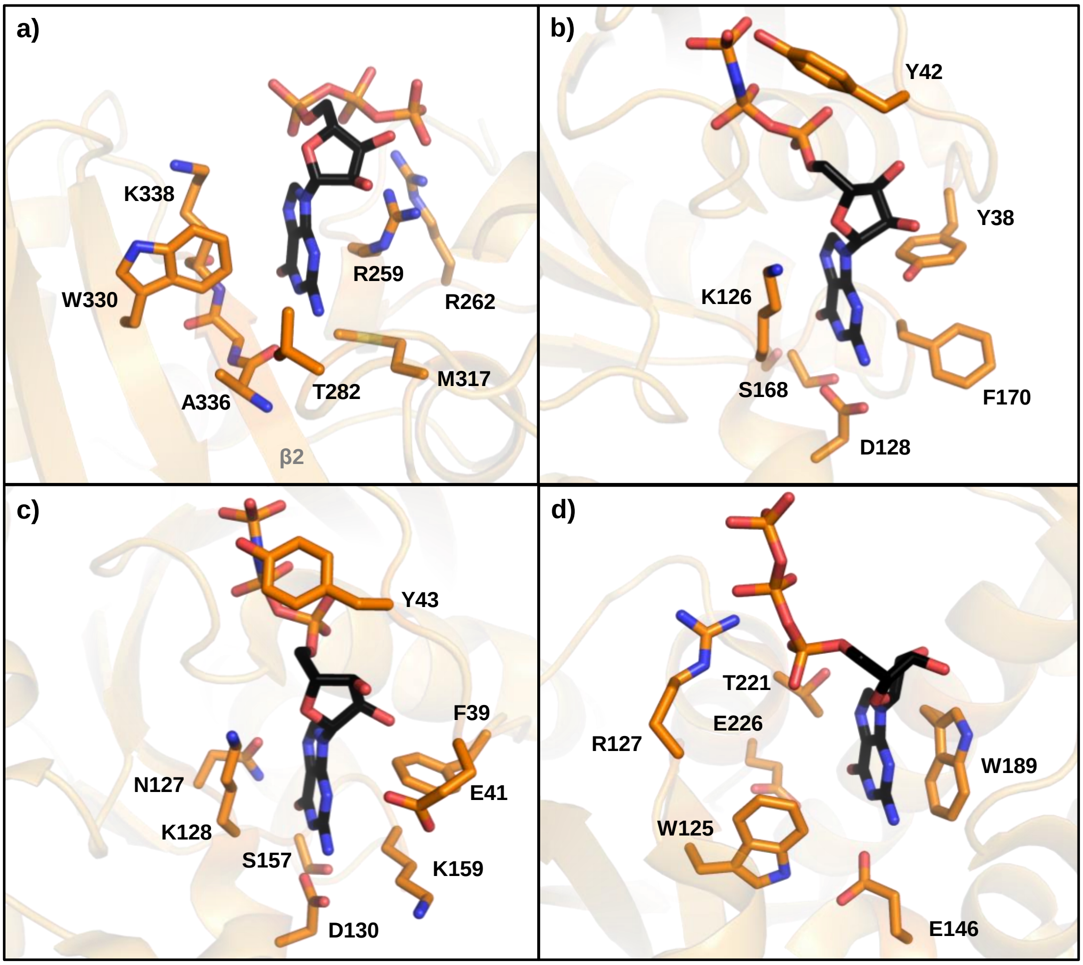

Supplementary Figure 7. Comparatisión of the GTP binding site of N^{CTD} with structurally symmilar human proteins. a) Close view of the GTP binding site of of SARS-CoV-2 N^{CTD}, **b)** Human Rho-related GTP-binding protein Rho6 in complex with GNP (PDB ID: 2REX), **c)** Human Ras related protein Ral-A in complex with GNP (PDB ID: 1ZC3), **d)** Human CD38 in complex with GTP (PDB ID: 3DZI). The residues that mediate interaction with the nucleotide are represented in sticks with carbon atoms coloured in orange. Ligands are represented in sticks, with carbon in black. Nitrogen, oxygen and phosphorous atoms are colored in blue, red and orange, respectively.

Supplementary Table 1: Interactions between GTP and SARS-CoV-2 N^{CTD}.

Residues of N^{CTD} subunits are colored in blue and orange, and the symmetric molecule in grey.

GTP		SARS-CoV-2 N^{CTD}		
Moiety	Atom	Atom	Residue	Distance (Å)
Guanine	O6	N	K338	2.86
	C6	CB	R259	4.29
		CD		3.45
		CE	M317	3.63
		CZ3	W330	3.88
		CH2		4.54
		CE3		4.65
		C	A336	4.87
		CA	I337	4.18
		C		4.32
		CA	K338	4.35
		CB		3.94
	N1	O	A336	2.95
	C2	CE	M317	3.68
		CD	R259	4.03
		CG2	T282	4.09
		CH2		4.34
		CH2	W330	4.34
		CZ3		3.67
		CE3		4.64
	N2	O	A336	2.95
		O	T282	3.59
	C4	CD	R259	3.63
		CZ		4.36
		CZ2	W330	4.99
		CZ3		3.85
		CH2		3.80
	C5	CB	M317	4.88
		CD	K338	4.87
		CB		3.93
		CZ3	W330	3.96
		CH2		4.15
		CG	R259	4.55
		CD		3.34
		CZ		4.79
		CB		4.50
	C8	CD	K338	4.42
		CB		4.60
		CZ3	W330	5.03
		CH2		4.60
		CD	R259	4.23
		CZ		4.84
Ribose	C1	C	T362	3.81
		CA		3.56
		CB		3.69
		CG2		4.30
		CH2	W330	4.66
		CD	R259	5.06
		CZ		4.60
	C2	CD		4.56
		CZ		3.44
	O2	NE		4.53
		NH1		3.65
		NH2		4.37
	C3	CZ		3.76
		CG2	T362	4.34
	O3	NH1	R259	3.82
		NH2		3.52
	C4	CZ		5.4

Conformation A				
GTP		SARS-CoV-2 N^{CTD}		
Moiety	Atom	Atom	Residue	Distance (Å)
Pα	O1A	NZ	K338	4.22
	O2A			4.74
Pβ	O1B	NH1	R262	4.89
		NH2		3.39
	O2B	NH2	R262	4.65
	O3B	NH2	R259	3.58
		NH1	R262	4.26
		NH2		4.07
Pγ	O1G	NH2	R259	4.64
		NE	R262	4.97
		NH1		3.40
		NH2		3.22
	O2G	NH2	R259	4.85
	OG3	NH1		3.98
		NH2		2.67
		NH1	R262	3.32
		NH2		4.42

Conformation B				
GTP		SARS-CoV-2 N^{CTD}		
Moiety	Atom	Atom	Residue	Distance (Å)
Pα	O1A	NH2	R259	4.95
	O3A	NH2		3.80
		NH2	R262	4.04
Pβ	O1B	NH2	R259	4.25
	O2B	NH2		3.47
		NE	R262	4.54
		NH1		2.62
		NH2		3.02
Pγ	O1G	NH2		3.52
		NE		4.97
		NH1		3.40
		NH2		3.22
	OG3	NH1		4.15
		NH2		3.34

Supplementary Table 2: Linear regression of quenching.

Linear regression	N^{CTD}	N^{CTD}+GTP	$N^{CTD-W330A}$	$N^{CTD-W330A}$+GTP
**Best-fit values**				
Slope	6,310 ± 0,08360	5,173 ± 0,09601	1,990 ± 0,06165	1,738 ± 0,05859
Y-intercept when X=0.0	1,077 ± 0,05585	0,9515 ± 0,06145	0,8802 ± 0,03946	1,040 ± 0,03750
X-intercept when Y=0.0	-0,1708	-0,1839	-0,4422	-0,5984
1/slope	0,1585	0,1933	0,5024	0,5755
**95% Confidence Intervals**				
Slope	6,146 to 6,473	4,982 to 5,364	1,867 to 2,113	1,621 to 1,854
Y-intercept when X=0.0	0,9679 to 1,187	0,8290 to 1,074	0,8016 to 0,9588	0,9651 to 1,115
X-intercept when Y=0.0	-0,1918 to -0,1505	-0,2135 to -0,1560	-0,5071 to -0,3841	-0,6795 to -0,5266
**Goodness of Fit**				
R square	0,976	0,9722	0,9262	0,9138
Sy.x	0,488	0,4182	0,2685	0,2552
**Data**				
Number of X values	21	21	21	21
Maximum number of Y replicate	10	7	7	7
Total number of values	142	85	85	85
Number of missing values	68	125	125	125
Equation	Y = 6,310*X + 1,077	Y = 5,173*X + 0,9515	Y = 1,990*X + 0,8802	Y = 1,738*X + 1,040

Linear regression	N^{CTD}+ATP	N^{CTD}+CTP	N^{CTD}+UTP
**Best-fit values**			
Slope	6,593 ± 0,1503	5,725 ± 0,1087	6,163 ± 0,1606
Y-intercept when X=0.0	0,9751 ± 0,1064	1,127 ± 0,07695	0,8585 ± 0,1101
X-intercept when Y=0.0	-0,1479	-0,1969	-0,1393
1/slope	0,1517	0,1747	0,1623
**95% Confidence Intervals**			
Slope	6,292 to 6,894	5,507 to 5,943	5,840 to 6,485
Y-intercept when X=0.0	0,7618 to 1,188	0,9728 to 1,281	0,6377 to 1,079
X-intercept when Y=0.0	-0,1866 to -0,1118	-0,2305 to -0,1653	-0,1822 to -0,09972
**Goodness of Fit**			
R square	0,9722	0,9805	0,9646
Sy.x	0,5817	0,4209	0,5997
**Data**			
Number of X values	19	19	19
Maximum number of Y replicate	3	3	3
Total number of values	57	57	56
Number of missing values	153	153	154
Equation	Y = 6,593*X + 0,9751	Y = 5,725*X + 1,127	Y = 6,163*X + 0,8585

Supplementary Table 3: Comparison of quenching slopes.

ANOVA table	SS	DF	MS	F (DFn, DFd)	P value
Treatment (between columns)	159,4	6	26,56	F (6, 33) = 527,0	P < 0,0001
Residual (within columns)	1,663	33	0,0504		
Total	161	39			

Tukey's multiple comparisons test	Mean Diff,	95% CI of diff,	Significant	Summary	Adjusted P Value
N^{CTD} vs. N^{CTD}+GTP	1,137	0,7899 to 1,484	Yes	****	< 0,0001
N^{CTD} vs. N^{CTD}+ATP	-0,283	-0,7466 to 0,1806	No	ns	0,4852
N^{CTD} vs. N^{CTD}+CTP	0,585	0,1214 to 1,049	Yes	**	0,0063
N^{CTD} vs. N^{CTD}+UTP	0,147	-0,3166 to 0,6106	No	ns	0,9516
$N^{CTD-W330A}$ vs. $N^{CTD-W330A}$+GTP	0,252	-0,1245 to 0,6285	No	ns	0,3761

Test details	Mean 1	Mean 2	Mean Diff,	SE of diff,	n1	n2	q	DF
N^{CTD} vs. N^{CTD}+GTP	6,31	5,173	1,137	0,1106	10	7	14,53	33
N^{CTD} vs. N^{CTD}+ATP	6,31	6,593	-0,283	0,1478	10	3	2,708	33
N^{CTD} vs. N^{CTD}+CTP	6,31	5,725	0,585	0,1478	10	3	5,598	33
N^{CTD} vs. N^{CTD}+UTP	6,31	6,163	0,147	0,1478	10	3	1,407	33
$N^{CTD-W330A}$ vs. $N^{CTD-W330A}$+GTP	1,99	1,738	0,252	0,12	7	7	2,97	33

Supplementary Table 4: Statistical data of DSF and MST experiments.

Boltzmann Sigmoidal fitting	Tm ± S.E.	Slope ± S.E.	R^2
N^{CTD}	48.96 ± 0.03	0.79 ± 0.03	0.99
N^{CTD} + GTP	49.97 ± 0.03	0.81 ± 0.02	0.99
$N^{CTD-W330A}$	47.98 ± 0.07	2.18 ± 0.07	0.99
$N^{CTD-W330A}$ + GTP	47.67 ± 0.07	2.07 ± 0.07	0.99

MicroScale Thermophoresis	N^{CTD}	$N^{CTD-W330A}$
Target Concentration:	20nM	20nM
Ligand Name	GTP	GTP
Ligand Concentration	50 mM to 0.00153 mM	50 mM to 0.00153 mM
n:	3	3
Excitation Power:	77%	77%
MST Power:	40%	40%
Temperature:	25ºC	25ºC
Kd (M):	0.00019642	0.00085815
Kd Confidence:	[0.00012558 - 0.00030723]	[0.00057568 - 0.0012792]
Response Amplitude:	13.131	13.135
Unbound:	939.97	904.66
Bound:	926.84	899.98
St. Error of Regression	0.981	0.853
Signal to noise:	14.367	16.538
