# Supplementary material for: Identification of a guanine-specific pocket in the protein N of SARS-CoV-2
Source: Commun Biol. 2022 Jul 16;5:711. doi: 10.1038/s42003-022-03647-8 (PMC9288159; doi:10.1038/s42003-022-03647-8)
Supplement: Supplementary file 3 — Description of Additional Supplementary Files [file 42003_2022_3647_MOESM3_ESM.pdf]

Description of Additional Supplementary Files

File name: Supplementary Data 1

Description: PDB validation report for 7O05

File name: Supplementary Data 2

Description: PDB validation report for 7O35

File name: Supplementary Data 3

Description: PDB validation report for 7O36
