# Supplementary material for: Identification of a guanine-specific pocket in the protein N of SARS-CoV-2
Source: Commun Biol. 2022 Jul 16;5:711. doi: 10.1038/s42003-022-03647-8 (PMC9288159; doi:10.1038/s42003-022-03647-8)
Supplement: Supplementary file 4 — Supplementary Data 1 [file 42003_2022_3647_MOESM4_ESM.pdf]

Full wwPDB X-ray Structure Validation Report (i)

Mar 29, 2021 – 07:59 pm BST

PDB ID	:	7O05
Title	:	Crystal structure of SARS-CoV-2 N-CTD
Deposited on	:	2021-03-25
Resolution	:	1.94 Å(reported)

This is a Full wwPDB X-ray Structure Validation Report.

This report is produced by the wwPDB biocuration pipeline after annotation of the structure.

We welcome your comments at *validation@mail.wwpdb.org*
A user guide is available at
https://www.wwpdb.org/validation/2017/XrayValidationReportHelp
with specific help available everywhere you see the (i) symbol.

CONFIDENTIAL VALIDATION REPORT

The following versions of software and data (see references (i)) were used in the production of this report:

MolProbity	:	4.02b-467
Xtriage (Phenix)	:	1.13
EDS	:	2.18
Percentile statistics	:	20191225.v01 (using entries in the PDB archive December 25th 2019)
Refmac	:	5.8.0158
CCP4	:	7.0.044 (Gargrove)
Ideal geometry (proteins)	:	Engh & Huber (2001)
Ideal geometry (DNA, RNA)	:	Parkinson et al. (1996)
Validation Pipeline (wwPDB-VP)	:	2.18

1 Overall quality at a glance (i)

The following experimental techniques were used to determine the structure:
X-RAY DIFFRACTION

The reported resolution of this entry is 1.94 Å.

Percentile scores (ranging between 0-100) for global validation metrics of the entry are shown in the following graphic. The table shows the number of entries on which the scores are based.

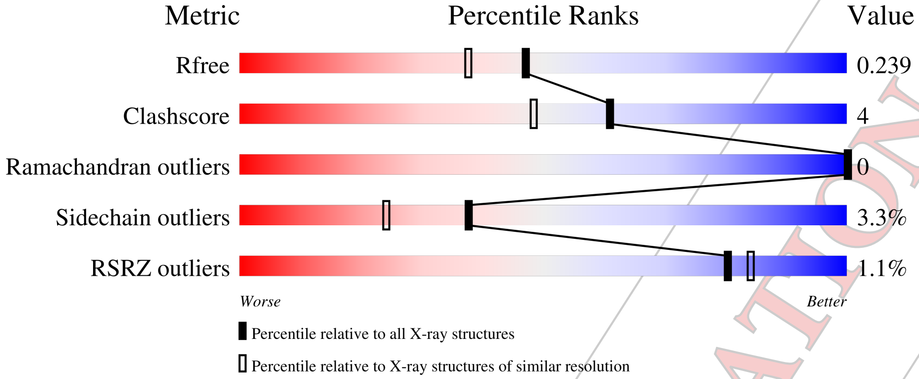

Metric	Whole archive (#Entries)	Similar resolution (#Entries, resolution range(Å))
R_{free}	130704	4310 (1.96-1.92)
Clashscore	141614	1023 (1.94-1.94)
Ramachandran outliers	138981	1007 (1.94-1.94)
Sidechain outliers	138945	1007 (1.94-1.94)
RSRZ outliers	127900	4250 (1.96-1.92)

The table below summarises the geometric issues observed across the polymeric chains and their fit to the electron density. The red, orange, yellow and green segments of the lower bar indicate the fraction of residues that contain outliers for >=3, 2, 1 and 0 types of geometric quality criteria respectively. A grey segment represents the fraction of residues that are not modelled. The numeric value for each fraction is indicated below the corresponding segment, with a dot representing fractions <=5% The upper red bar (where present) indicates the fraction of residues that have poor fit to the electron density. The numeric value is given above the bar.

Mol	Chain	Length	Quality of chain
1	A	136	75% • • 20%
1	B	136	% 71% 10% 20%
1	C	136	% 75% 7% 18%
1	D	136	% 68% 13% 18%

CONFIDENTIAL VALIDATION REPORT

2 Entry composition (i)

There are 2 unique types of molecules in this entry. The entry contains 3938 atoms, of which 0 are hydrogens and 0 are deuteriums.

In the tables below, the ZeroOcc column contains the number of atoms modelled with zero occupancy, the AltConf column contains the number of residues with at least one atom in alternate conformation and the Trace column contains the number of residues modelled with at most 2 atoms.

- Molecule 1 is a protein called Nucleoprotein.

Mol	Chain	Residues	Atoms					ZeroOcc	AltConf	Trace
1	A	109	Total 894	C 568	N 161	O 163	S 2	0	3	0
1	B	109	Total 894	C 568	N 161	O 163	S 2	0	2	0
1	C	111	Total 899	C 569	N 163	O 165	S 2	0	2	0
1	D	111	Total 905	C 574	N 163	O 166	S 2	0	2	0

There are 72 discrepancies between the modelled and reference sequences:

Chain	Residue	Modelled	Actual	Comment	Reference
A	229	MET	-	initiating methionine	UNP P0DTC9
A	230	GLY	-	expression tag	UNP P0DTC9
A	231	SER	-	expression tag	UNP P0DTC9
A	232	SER	-	expression tag	UNP P0DTC9
A	233	HIS	-	expression tag	UNP P0DTC9
A	234	HIS	-	expression tag	UNP P0DTC9
A	235	HIS	-	expression tag	UNP P0DTC9
A	236	HIS	-	expression tag	UNP P0DTC9
A	237	HIS	-	expression tag	UNP P0DTC9
A	238	HIS	-	expression tag	UNP P0DTC9
A	239	GLY	-	expression tag	UNP P0DTC9
A	240	GLU	-	expression tag	UNP P0DTC9
A	241	ASN	-	expression tag	UNP P0DTC9
A	242	LEU	-	expression tag	UNP P0DTC9
A	243	TYR	-	expression tag	UNP P0DTC9
A	244	PHE	-	expression tag	UNP P0DTC9
A	245	GLN	-	expression tag	UNP P0DTC9
A	246	SER	-	expression tag	UNP P0DTC9
B	229	MET	-	initiating methionine	UNP P0DTC9
B	230	GLY	-	expression tag	UNP P0DTC9
B	231	SER	-	expression tag	UNP P0DTC9

Continued on next page...

CONFIDENTIAL VALIDATION REPORT

Continued from previous page...

Chain	Residue	Modelled	Actual	Comment	Reference
B	232	SER	-	expression tag	UNP P0DTC9
B	233	HIS	-	expression tag	UNP P0DTC9
B	234	HIS	-	expression tag	UNP P0DTC9
B	235	HIS	-	expression tag	UNP P0DTC9
B	236	HIS	-	expression tag	UNP P0DTC9
B	237	HIS	-	expression tag	UNP P0DTC9
B	238	HIS	-	expression tag	UNP P0DTC9
B	239	GLY	-	expression tag	UNP P0DTC9
B	240	GLU	-	expression tag	UNP P0DTC9
B	241	ASN	-	expression tag	UNP P0DTC9
B	242	LEU	-	expression tag	UNP P0DTC9
B	243	TYR	-	expression tag	UNP P0DTC9
B	244	PHE	-	expression tag	UNP P0DTC9
B	245	GLN	-	expression tag	UNP P0DTC9
B	246	SER	-	expression tag	UNP P0DTC9
C	229	MET	-	initiating methionine	UNP P0DTC9
C	230	GLY	-	expression tag	UNP P0DTC9
C	231	SER	-	expression tag	UNP P0DTC9
C	232	SER	-	expression tag	UNP P0DTC9
C	233	HIS	-	expression tag	UNP P0DTC9
C	234	HIS	-	expression tag	UNP P0DTC9
C	235	HIS	-	expression tag	UNP P0DTC9
C	236	HIS	-	expression tag	UNP P0DTC9
C	237	HIS	-	expression tag	UNP P0DTC9
C	238	HIS	-	expression tag	UNP P0DTC9
C	239	GLY	-	expression tag	UNP P0DTC9
C	240	GLU	-	expression tag	UNP P0DTC9
C	241	ASN	-	expression tag	UNP P0DTC9
C	242	LEU	-	expression tag	UNP P0DTC9
C	243	TYR	-	expression tag	UNP P0DTC9
C	244	PHE	-	expression tag	UNP P0DTC9
C	245	GLN	-	expression tag	UNP P0DTC9
C	246	SER	-	expression tag	UNP P0DTC9
D	229	MET	-	initiating methionine	UNP P0DTC9
D	230	GLY	-	expression tag	UNP P0DTC9
D	231	SER	-	expression tag	UNP P0DTC9
D	232	SER	-	expression tag	UNP P0DTC9
D	233	HIS	-	expression tag	UNP P0DTC9
D	234	HIS	-	expression tag	UNP P0DTC9
D	235	HIS	-	expression tag	UNP P0DTC9
D	236	HIS	-	expression tag	UNP P0DTC9
D	237	HIS	-	expression tag	UNP P0DTC9

Continued on next page...

CONFIDENTIAL VALIDATION REPORT

Continued from previous page...

Chain	Residue	Modelled	Actual	Comment	Reference
D	238	HIS	-	expression tag	UNP P0DTC9
D	239	GLY	-	expression tag	UNP P0DTC9
D	240	GLU	-	expression tag	UNP P0DTC9
D	241	ASN	-	expression tag	UNP P0DTC9
D	242	LEU	-	expression tag	UNP P0DTC9
D	243	TYR	-	expression tag	UNP P0DTC9
D	244	PHE	-	expression tag	UNP P0DTC9
D	245	GLN	-	expression tag	UNP P0DTC9
D	246	SER	-	expression tag	UNP P0DTC9

- Molecule 2 is water.

Mol	Chain	Residues	Atoms	ZeroOcc	AltConf
2	A	89	Total O<br>89 89	0	0
2	B	74	Total O<br>74 74	0	0
2	C	86	Total O<br>86 86	0	0
2	D	97	Total O<br>97 97	0	0

CONFIDENTIAL VALIDATION REPORT

3 Residue-property plots (i)

These plots are drawn for all protein, RNA, DNA and oligosaccharide chains in the entry. The first graphic for a chain summarises the proportions of the various outlier classes displayed in the second graphic. The second graphic shows the sequence view annotated by issues in geometry and electron density. Residues are color-coded according to the number of geometric quality criteria for which they contain at least one outlier: green = 0, yellow = 1, orange = 2 and red = 3 or more. A red dot above a residue indicates a poor fit to the electron density (RSRZ > 2). Stretches of 2 or more consecutive residues without any outlier are shown as a green connector. Residues present in the sample, but not in the model, are shown in grey.

- Molecule 1: Nucleoprotein

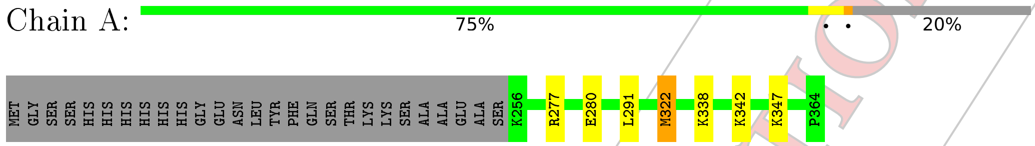

- Molecule 1: Nucleoprotein

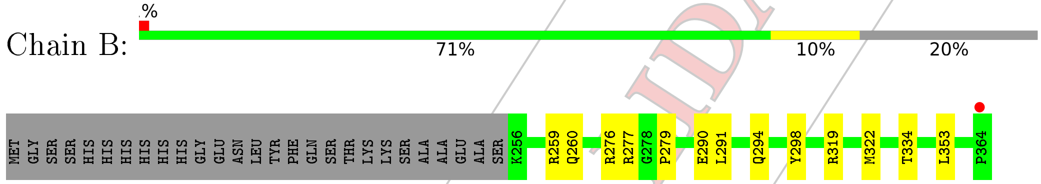

- Molecule 1: Nucleoprotein

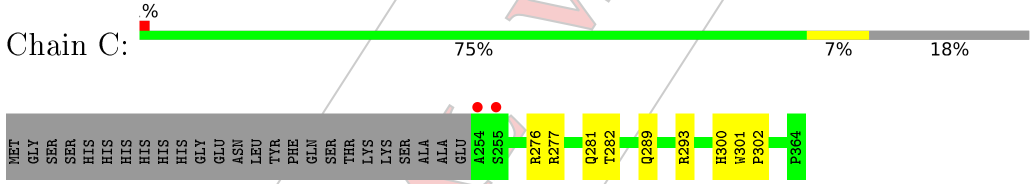

- Molecule 1: Nucleoprotein

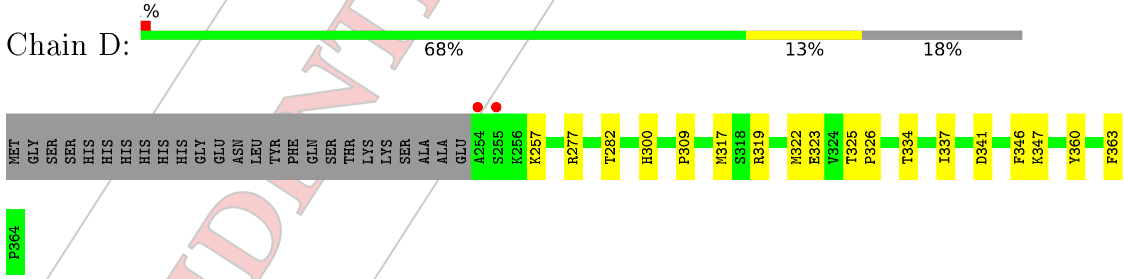

CONFIDENTIAL VALIDATION REPORT

4 Data and refinement statistics (i)

Property	Value	Source
Space group	P 1	Depositor
Cell constants<br>a, b, c, α, β, γ	43.84Å 44.82Å 59.12Å<br>92.04° 96.20° 90.00°	Depositor
Resolution (Å)	44.79 – 1.94<br>44.79 – 1.94	Depositor<br>EDS
% Data completeness<br>(in resolution range)	96.1 (44.79-1.94)<br>96.1 (44.79-1.94)	Depositor<br>EDS
R_{merge}	0.17	Depositor
R_{sym}	(Not available)	Depositor
$< I/\sigma(I) >$ [1]	2.04 (at 1.94Å)	Xtriage
Refinement program	REFMAC 5.8.0103	Depositor
R, R_{free}	0.177 , 0.233<br>0.185 , 0.239	Depositor<br>DCC
R_{free} test set	1635 reflections (5.10%)	wwPDB-VP
Wilson B-factor (Å^2)	22.6	Xtriage
Anisotropy	0.694	Xtriage
Bulk solvent k_{sol}(e/Å^3), B_{sol}(Å^2)	0.37 , 49.1	EDS
L-test for twinning[2]	$< \|L\| > = 0.51$, $< L^2 > = 0.34$	Xtriage
Estimated twinning fraction	0.023 for -h,k,-l	Xtriage
F_o,F_c correlation	0.96	EDS
Total number of atoms	3938	wwPDB-VP
Average B, all atoms (Å^2)	28.0	wwPDB-VP

Xtriage's analysis on translational NCS is as follows: *The largest off-origin peak in the Patterson function is 11.71% of the height of the origin peak. No significant pseudotranslation is detected.*

[1] Intensities estimated from amplitudes.

[2] Theoretical values of $< |L| >$, $< L^2 >$ for acentric reflections are 0.5, 0.333 respectively for untwinned datasets, and 0.375, 0.2 for perfectly twinned datasets.

CONFIDENTIAL VALIDATION REPORT

5 Model quality (i)

5.1 Standard geometry (i)

The Z score for a bond length (or angle) is the number of standard deviations the observed value is removed from the expected value. A bond length (or angle) with $|Z| > 5$ is considered an outlier worth inspection. RMSZ is the root-mean-square of all Z scores of the bond lengths (or angles).

Mol	Chain	Bond lengths		Bond angles	
		RMSZ	#$\|Z\|$ >5	RMSZ	#$\|Z\|$ >5
1	A	0.83	0/917	0.89	0/1239
1	B	0.87	0/917	0.93	0/1236
1	C	0.88	0/922	0.90	0/1244
1	D	0.94	0/928	0.89	0/1251
All	All	0.88	0/3684	0.90	0/4970

There are no bond length outliers.

There are no bond angle outliers.

There are no chirality outliers.

There are no planarity outliers.

5.2 Too-close contacts (i)

In the following table, the Non-H and H(model) columns list the number of non-hydrogen atoms and hydrogen atoms in the chain respectively. The H(added) column lists the number of hydrogen atoms added and optimized by MolProbity. The Clashes column lists the number of clashes within the asymmetric unit, whereas Symm-Clashes lists symmetry-related clashes.

Mol	Chain	Non-H	H(model)	H(added)	Clashes	Symm-Clashes
1	A	894	0	871	6	0
1	B	894	0	881	8	0
1	C	899	0	875	10	0
1	D	905	0	891	11	0
2	A	89	0	0	3	0
2	B	74	0	0	0	0
2	C	86	0	0	4	0
2	D	97	0	0	3	0
All	All	3938	0	3518	32	0

The all-atom clashscore is defined as the number of clashes found per 1000 atoms (including hydrogen atoms). The all-atom clashscore for this structure is 4.

CONFIDENTIAL VALIDATION REPORT

All (32) close contacts within the same asymmetric unit are listed below, sorted by their clash magnitude.

Atom-1	Atom-2	Interatomic distance (Å)	Clash overlap (Å)
1:C:289[B]:GLN:O	1:C:293[B]:ARG:HG3	1.78	0.83
1:D:337[B]:ILE:O	1:D:337[B]:ILE:HG13	1.81	0.79
1:A:291[B]:LEU:C	1:A:291[B]:LEU:HD23	2.18	0.64
1:C:281:GLN:O	1:C:282:THR:HG22	2.01	0.60
1:C:276:ARG:NE	2:C:402:HOH:O	2.37	0.57
1:D:319:ARG:HB2	1:D:334:THR:HG22	1.89	0.54
1:B:290:GLU:O	1:B:294:GLN:HG3	2.07	0.54
1:B:291[B]:LEU:C	1:B:291[B]:LEU:HD13	2.31	0.51
1:B:319:ARG:HB2	1:B:334:THR:HG22	1.93	0.51
1:A:291[B]:LEU:HD23	1:A:291[B]:LEU:O	2.10	0.50
1:A:338:LYS:NZ	2:A:402:HOH:O	2.44	0.50
1:B:291[B]:LEU:HD23	1:B:298:TYR:CD2	2.46	0.50
1:C:289[B]:GLN:O	1:C:293[B]:ARG:CG	2.56	0.49
1:D:347:LYS:NZ	2:D:402:HOH:O	2.32	0.47
1:C:293[B]:ARG:HG3	1:C:293[B]:ARG:H	1.55	0.47
1:D:300:HIS:ND1	2:D:404:HOH:O	2.35	0.47
1:A:322:MET:HA	1:A:322:MET:HE2	1.95	0.47
1:A:347:LYS:CE	2:A:401:HOH:O	2.63	0.47
1:D:323:GLU:HG2	2:D:447:HOH:O	2.15	0.45
1:C:282:THR:HG23	2:C:404:HOH:O	2.16	0.45
1:B:260:GLN:O	1:D:309:PRO:HA	2.18	0.43
1:C:300:HIS:ND1	2:C:403:HOH:O	2.37	0.43
1:A:347:LYS:NZ	2:A:401:HOH:O	2.43	0.42
1:C:281:GLN:O	1:C:282:THR:CG2	2.65	0.42
1:D:360:TYR:HA	1:D:363:PHE:CZ	2.55	0.42
1:B:291[A]:LEU:HD12	1:B:291[A]:LEU:O	2.20	0.41
1:B:259[A]:ARG:CZ	1:D:317:MET:HG2	2.50	0.41
1:D:341:ASP:HA	1:D:346:PHE:CD1	2.56	0.41
1:D:325:THR:HB	1:D:326:PRO:HD2	2.03	0.41
1:C:301:TRP:N	1:C:302:PRO:CD	2.84	0.41
1:C:276:ARG:CZ	2:C:402:HOH:O	2.69	0.41
1:B:353:LEU:HD13	1:D:322:MET:HE3	2.02	0.40

There are no symmetry-related clashes.

CONFIDENTIAL VALIDATION REPORT

5.3 Torsion angles (i)

5.3.1 Protein backbone (i)

In the following table, the Percentiles column shows the percent Ramachandran outliers of the chain as a percentile score with respect to all X-ray entries followed by that with respect to entries of similar resolution.

The Analysed column shows the number of residues for which the backbone conformation was analysed, and the total number of residues.

Mol	Chain	Analysed	Favoured	Allowed	Outliers	Percentiles	
1	A	110/136 (81%)	109 (99%)	1 (1%)	0	100	100
1	B	109/136 (80%)	106 (97%)	3 (3%)	0	100	100
1	C	111/136 (82%)	107 (96%)	4 (4%)	0	100	100
1	D	111/136 (82%)	110 (99%)	1 (1%)	0	100	100
All	All	441/544 (81%)	432 (98%)	9 (2%)	0	100	100

There are no Ramachandran outliers to report.

5.3.2 Protein sidechains (i)

In the following table, the Percentiles column shows the percent sidechain outliers of the chain as a percentile score with respect to all X-ray entries followed by that with respect to entries of similar resolution.

The Analysed column shows the number of residues for which the sidechain conformation was analysed, and the total number of residues.

Mol	Chain	Analysed	Rotameric	Outliers	Percentiles	
1	A	92/113 (81%)	88 (96%)	4 (4%)	29	14
1	B	93/113 (82%)	89 (96%)	4 (4%)	29	14
1	C	92/113 (81%)	91 (99%)	1 (1%)	73	67
1	D	94/113 (83%)	91 (97%)	3 (3%)	39	25
All	All	371/452 (82%)	359 (97%)	12 (3%)	38	25

All (12) residues with a non-rotameric sidechain are listed below:

Mol	Chain	Res	Type
1	A	277	ARG
1	A	280	GLU

Continued on next page...

CONFIDENTIAL VALIDATION REPORT

Continued from previous page...

Mol	Chain	Res	Type
1	A	322	MET
1	A	342	LYS
1	B	276	ARG
1	B	277	ARG
1	B	279	PRO
1	B	322	MET
1	C	277	ARG
1	D	257	LYS
1	D	277	ARG
1	D	282	THR

Sometimes sidechains can be flipped to improve hydrogen bonding and reduce clashes. All (4) such sidechains are listed below:

Mol	Chain	Res	Type
1	A	289	GLN
1	B	294	GLN
1	C	294	GLN
1	D	294	GLN

5.3.3 RNA (i)

There are no RNA molecules in this entry.

5.4 Non-standard residues in protein, DNA, RNA chains (i)

There are no non-standard protein/DNA/RNA residues in this entry.

5.5 Carbohydrates (i)

There are no monosaccharides in this entry.

5.6 Ligand geometry (i)

There are no ligands in this entry.

5.7 Other polymers (i)

There are no such residues in this entry.

CONFIDENTIAL VALIDATION REPORT

5.8 Polymer linkage issues (i)

There are no chain breaks in this entry.

CONFIDENTIAL VALIDATION REPORT

6 Fit of model and data (i)

6.1 Protein, DNA and RNA chains (i)

In the following table, the column labelled '#RSRZ> 2' contains the number (and percentage) of RSRZ outliers, followed by percent RSRZ outliers for the chain as percentile scores relative to all X-ray entries and entries of similar resolution. The OWAB column contains the minimum, median, 95th percentile and maximum values of the occupancy-weighted average B-factor per residue. The column labelled 'Q< 0.9' lists the number of (and percentage) of residues with an average occupancy less than 0.9.

Mol	Chain	Analysed	<RSRZ>	#RSRZ>2			OWAB(Å^2)	Q<0.9
1	A	109/136 (80%)	-0.06	0	100	100	15, 25, 42, 62	0
1	B	109/136 (80%)	-0.11	1 (0%)	84	87	15, 26, 46, 64	0
1	C	111/136 (81%)	-0.14	2 (1%)	68	74	16, 24, 42, 65	0
1	D	111/136 (81%)	-0.08	2 (1%)	68	74	15, 24, 45, 64	0
All	All	440/544 (80%)	-0.10	5 (1%)	80	84	15, 25, 46, 65	0

All (5) RSRZ outliers are listed below:

Mol	Chain	Res	Type	RSRZ
1	D	254	ALA	4.5
1	D	255	SER	3.5
1	C	254	ALA	3.0
1	C	255	SER	2.7
1	B	364	PRO	2.4

6.2 Non-standard residues in protein, DNA, RNA chains (i)

There are no non-standard protein/DNA/RNA residues in this entry.

6.3 Carbohydrates (i)

There are no monosaccharides in this entry.

6.4 Ligands (i)

There are no ligands in this entry.

CONFIDENTIAL VALIDATION REPORT

6.5 Other polymers (i)

There are no such residues in this entry.

CONFIDENTIAL VALIDATION REPORT
