# Supplementary material for: Identification of a guanine-specific pocket in the protein N of SARS-CoV-2
Source: Commun Biol. 2022 Jul 16;5:711. doi: 10.1038/s42003-022-03647-8 (PMC9288159; doi:10.1038/s42003-022-03647-8)
Supplement: Supplementary file 5 — Supplementary Data 2 [file 42003_2022_3647_MOESM5_ESM.pdf]

Full wwPDB X-ray Structure Validation Report (i)

Apr 1, 2021 – 07:22 pm BST

PDB ID	:	7O35
Title	:	Crystal Structure of SARS-CoV-2 N-CTD in complex with GTP (I)
Deposited on	:	2021-04-01
Resolution	:	1.80 Å(reported)

This is a Full wwPDB X-ray Structure Validation Report.

This report is produced by the wwPDB biocuration pipeline after annotation of the structure.

We welcome your comments at *validation@mail.wwpdb.org*
A user guide is available at
https://www.wwpdb.org/validation/2017/XrayValidationReportHelp
with specific help available everywhere you see the (i) symbol.

The following versions of software and data (see references (i)) were used in the production of this report:

MolProbity	:	4.02b-467
Mogul	:	1.8.5 (274361), CSD as541be (2020)
Xtriage (Phenix)	:	1.13
EDS	:	2.18
buster-report	:	1.1.7 (2018)
Percentile statistics	:	20191225.v01 (using entries in the PDB archive December 25th 2019)
Refmac	:	5.8.0158
CCP4	:	7.0.044 (Gargrove)
Ideal geometry (proteins)	:	Engh & Huber (2001)
Ideal geometry (DNA, RNA)	:	Parkinson et al. (1996)
Validation Pipeline (wwPDB-VP)	:	2.18

CONFIDENTIAL VALIDATION REPORT

1 Overall quality at a glance (i)

The following experimental techniques were used to determine the structure:
X-RAY DIFFRACTION

The reported resolution of this entry is 1.80 Å.

Percentile scores (ranging between 0-100) for global validation metrics of the entry are shown in the following graphic. The table shows the number of entries on which the scores are based.

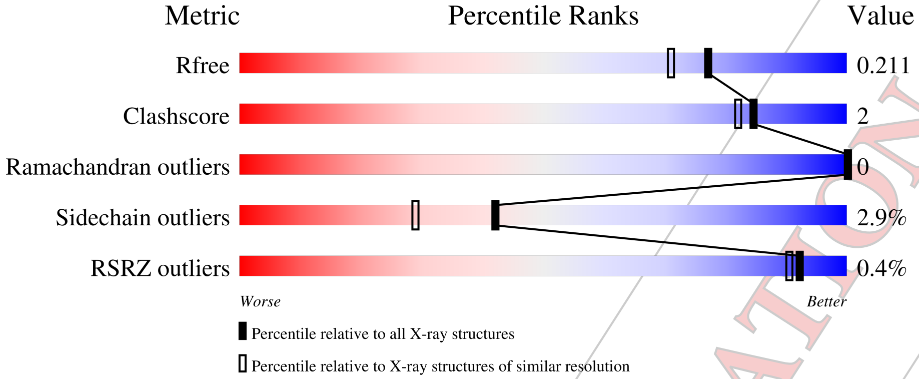

Metric	Whole archive (#Entries)	Similar resolution (#Entries, resolution range(Å))
R_{free}	130704	5950 (1.80-1.80)
Clashscore	141614	6793 (1.80-1.80)
Ramachandran outliers	138981	6697 (1.80-1.80)
Sidechain outliers	138945	6696 (1.80-1.80)
RSRZ outliers	127900	5850 (1.80-1.80)

The table below summarises the geometric issues observed across the polymeric chains and their fit to the electron density. The red, orange, yellow and green segments of the lower bar indicate the fraction of residues that contain outliers for >=3, 2, 1 and 0 types of geometric quality criteria respectively. A grey segment represents the fraction of residues that are not modelled. The numeric value for each fraction is indicated below the corresponding segment, with a dot representing fractions <=5% The upper red bar (where present) indicates the fraction of residues that have poor fit to the electron density. The numeric value is given above the bar.

Mol	Chain	Length	Quality of chain
1	A	136	76% • • 18%
1	B	136	84% 6% • 10%
1	C	136	% 71% 9% • 18%
1	D	136	% 73% 7% • 20%

CONFIDENTIAL VALIDATION REPORT

2 Entry composition (i)

There are 3 unique types of molecules in this entry. The entry contains 4146 atoms, of which 0 are hydrogens and 0 are deuteriums.

In the tables below, the ZeroOcc column contains the number of atoms modelled with zero occupancy, the AltConf column contains the number of residues with at least one atom in alternate conformation and the Trace column contains the number of residues modelled with at most 2 atoms.

- Molecule 1 is a protein called Nucleoprotein.

Mol	Chain	Residues	Atoms					ZeroOcc	AltConf	Trace
			Total	C	N	O	S			
1	A	111	904	572	163	167	2	0	2	0
1	B	123	982	624	173	183	2	0	0	0
1	C	111	893	566	159	166	2	0	1	0
1	D	109	893	566	161	164	2	0	2	0

There are 72 discrepancies between the modelled and reference sequences:

Chain	Residue	Modelled	Actual	Comment	Reference
A	229	MET	-	initiating methionine	UNP P0DTC9
A	230	GLY	-	expression tag	UNP P0DTC9
A	231	SER	-	expression tag	UNP P0DTC9
A	232	SER	-	expression tag	UNP P0DTC9
A	233	HIS	-	expression tag	UNP P0DTC9
A	234	HIS	-	expression tag	UNP P0DTC9
A	235	HIS	-	expression tag	UNP P0DTC9
A	236	HIS	-	expression tag	UNP P0DTC9
A	237	HIS	-	expression tag	UNP P0DTC9
A	238	HIS	-	expression tag	UNP P0DTC9
A	239	GLY	-	expression tag	UNP P0DTC9
A	240	GLU	-	expression tag	UNP P0DTC9
A	241	ASN	-	expression tag	UNP P0DTC9
A	242	LEU	-	expression tag	UNP P0DTC9
A	243	TYR	-	expression tag	UNP P0DTC9
A	244	PHE	-	expression tag	UNP P0DTC9
A	245	GLN	-	expression tag	UNP P0DTC9
A	246	SER	-	expression tag	UNP P0DTC9
B	229	MET	-	initiating methionine	UNP P0DTC9
B	230	GLY	-	expression tag	UNP P0DTC9
B	231	SER	-	expression tag	UNP P0DTC9

Continued on next page...

CONFIDENTIAL VALIDATION REPORT

Continued from previous page...

Chain	Residue	Modelled	Actual	Comment	Reference
B	232	SER	-	expression tag	UNP P0DTC9
B	233	HIS	-	expression tag	UNP P0DTC9
B	234	HIS	-	expression tag	UNP P0DTC9
B	235	HIS	-	expression tag	UNP P0DTC9
B	236	HIS	-	expression tag	UNP P0DTC9
B	237	HIS	-	expression tag	UNP P0DTC9
B	238	HIS	-	expression tag	UNP P0DTC9
B	239	GLY	-	expression tag	UNP P0DTC9
B	240	GLU	-	expression tag	UNP P0DTC9
B	241	ASN	-	expression tag	UNP P0DTC9
B	242	LEU	-	expression tag	UNP P0DTC9
B	243	TYR	-	expression tag	UNP P0DTC9
B	244	PHE	-	expression tag	UNP P0DTC9
B	245	GLN	-	expression tag	UNP P0DTC9
B	246	SER	-	expression tag	UNP P0DTC9
C	229	MET	-	initiating methionine	UNP P0DTC9
C	230	GLY	-	expression tag	UNP P0DTC9
C	231	SER	-	expression tag	UNP P0DTC9
C	232	SER	-	expression tag	UNP P0DTC9
C	233	HIS	-	expression tag	UNP P0DTC9
C	234	HIS	-	expression tag	UNP P0DTC9
C	235	HIS	-	expression tag	UNP P0DTC9
C	236	HIS	-	expression tag	UNP P0DTC9
C	237	HIS	-	expression tag	UNP P0DTC9
C	238	HIS	-	expression tag	UNP P0DTC9
C	239	GLY	-	expression tag	UNP P0DTC9
C	240	GLU	-	expression tag	UNP P0DTC9
C	241	ASN	-	expression tag	UNP P0DTC9
C	242	LEU	-	expression tag	UNP P0DTC9
C	243	TYR	-	expression tag	UNP P0DTC9
C	244	PHE	-	expression tag	UNP P0DTC9
C	245	GLN	-	expression tag	UNP P0DTC9
C	246	SER	-	expression tag	UNP P0DTC9
D	229	MET	-	initiating methionine	UNP P0DTC9
D	230	GLY	-	expression tag	UNP P0DTC9
D	231	SER	-	expression tag	UNP P0DTC9
D	232	SER	-	expression tag	UNP P0DTC9
D	233	HIS	-	expression tag	UNP P0DTC9
D	234	HIS	-	expression tag	UNP P0DTC9
D	235	HIS	-	expression tag	UNP P0DTC9
D	236	HIS	-	expression tag	UNP P0DTC9
D	237	HIS	-	expression tag	UNP P0DTC9

Continued on next page...

CONFIDENTIAL VALIDATION REPORT

Continued from previous page...

Chain	Residue	Modelled	Actual	Comment	Reference
D	238	HIS	-	expression tag	UNP P0DTC9
D	239	GLY	-	expression tag	UNP P0DTC9
D	240	GLU	-	expression tag	UNP P0DTC9
D	241	ASN	-	expression tag	UNP P0DTC9
D	242	LEU	-	expression tag	UNP P0DTC9
D	243	TYR	-	expression tag	UNP P0DTC9
D	244	PHE	-	expression tag	UNP P0DTC9
D	245	GLN	-	expression tag	UNP P0DTC9
D	246	SER	-	expression tag	UNP P0DTC9

- Molecule 2 is GUANOSINE-5'-TRIPHOSPHATE (three-letter code: GTP) (formula: $C_{10}H_{16}N_5O_{14}P_3$) (labeled as "Ligand of Interest" by depositor).

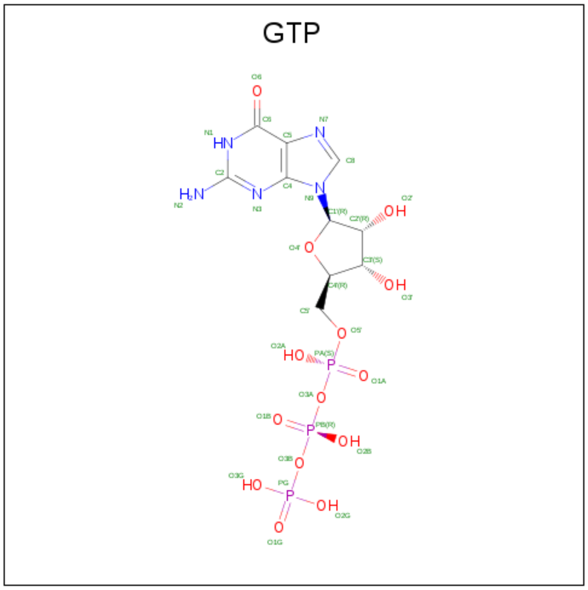

Mol	Chain	Residues	Atoms	ZeroOcc	AltConf
2	C	1	Total C N O P<br>32 10 5 14 3	0	0

- Molecule 3 is water.

Mol	Chain	Residues	Atoms	ZeroOcc	AltConf
3	A	101	Total O<br>101 101	0	0
3	B	126	Total O<br>126 126	0	0
3	C	106	Total O<br>106 106	0	0

Continued on next page...

CONFIDENTIAL VALIDATION REPORT

Continued from previous page...

Mol	Chain	Residues	Atoms		ZeroOcc	AltConf
			Total	O		
3	D	109	109	109	0	0

CONFIDENTIAL VALIDATION REPORT

3 Residue-property plots (i)

These plots are drawn for all protein, RNA, DNA and oligosaccharide chains in the entry. The first graphic for a chain summarises the proportions of the various outlier classes displayed in the second graphic. The second graphic shows the sequence view annotated by issues in geometry and electron density. Residues are color-coded according to the number of geometric quality criteria for which they contain at least one outlier: green = 0, yellow = 1, orange = 2 and red = 3 or more. A red dot above a residue indicates a poor fit to the electron density (RSRZ > 2). Stretches of 2 or more consecutive residues without any outlier are shown as a green connector. Residues present in the sample, but not in the model, are shown in grey.

- Molecule 1: Nucleoprotein

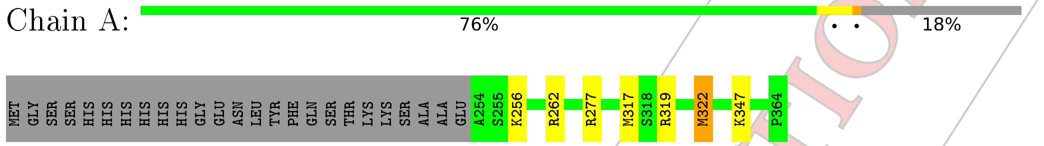

- Molecule 1: Nucleoprotein

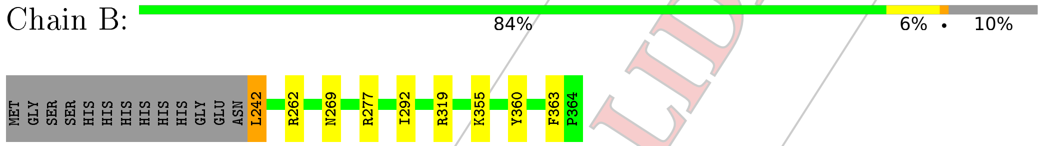

- Molecule 1: Nucleoprotein

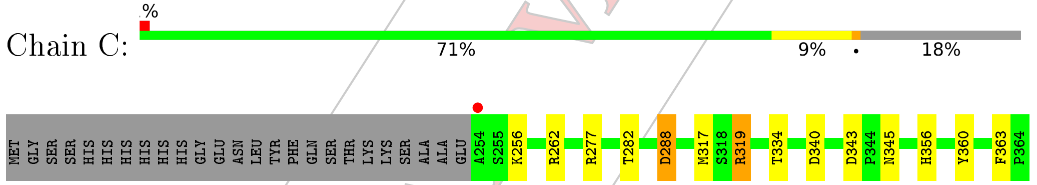

- Molecule 1: Nucleoprotein

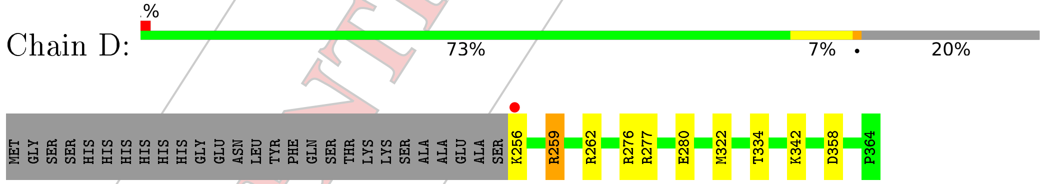

CONFIDENTIAL VALIDATION REPORT

4 Data and refinement statistics (i)

Property	Value	Source
Space group	P 1 21 1	Depositor
Cell constants<br>a, b, c, α, β, γ	43.77Å 92.72Å 68.73Å<br>90.00° 90.01° 90.00°	Depositor
Resolution (Å)	68.73 – 1.80<br>68.73 – 1.80	Depositor<br>EDS
% Data completeness<br>(in resolution range)	99.2 (68.73-1.80)<br>99.2 (68.73-1.80)	Depositor<br>EDS
R_{merge}	0.07	Depositor
R_{sym}	(Not available)	Depositor
$< I/\sigma(I) >$ [1]	5.87 (at 1.80Å)	Xtriage
Refinement program	REFMAC 5.8.0103	Depositor
R, R_{free}	0.156 , 0.203<br>0.165 , 0.211	Depositor<br>DCC
R_{free} test set	2511 reflections (4.98%)	wwPDB-VP
Wilson B-factor (Å^2)	21.2	Xtriage
Anisotropy	1.003	Xtriage
Bulk solvent k_{sol}(e/Å^3), B_{sol}(Å^2)	0.35 , 35.1	EDS
L-test for twinning[2]	$< \|L\| > = 0.45$, $< L^2 > = 0.28$	Xtriage
Estimated twinning fraction	0.114 for h,-k,-l	Xtriage
F_o,F_c correlation	0.97	EDS
Total number of atoms	4146	wwPDB-VP
Average B, all atoms (Å^2)	29.0	wwPDB-VP

Xtriage's analysis on translational NCS is as follows: *The largest off-origin peak in the Patterson function is 7.10% of the height of the origin peak. No significant pseudotranslation is detected.*

[1] Intensities estimated from amplitudes.

[2] Theoretical values of $< |L| >$, $< L^2 >$ for acentric reflections are 0.5, 0.333 respectively for untwinned datasets, and 0.375, 0.2 for perfectly twinned datasets.

CONFIDENTIAL VALIDATION REPORT

5 Model quality (i)

5.1 Standard geometry (i)

Bond lengths and bond angles in the following residue types are not validated in this section: GTP

The Z score for a bond length (or angle) is the number of standard deviations the observed value is removed from the expected value. A bond length (or angle) with $|Z| > 5$ is considered an outlier worth inspection. RMSZ is the root-mean-square of all Z scores of the bond lengths (or angles).

Mol	Chain	Bond lengths		Bond angles	
		RMSZ	#|Z| >5	RMSZ	#|Z| >5
1	A	0.99	0/927	1.02	4/1250 (0.3%)
1	B	0.99	0/1007	1.01	3/1357 (0.2%)
1	C	0.99	0/916	1.06	8/1236 (0.6%)
1	D	0.98	0/916	1.06	7/1235 (0.6%)
All	All	0.99	0/3766	1.04	22/5078 (0.4%)

There are no bond length outliers.

All (22) bond angle outliers are listed below:

Mol	Chain	Res	Type	Atoms	Z	Observed(°)	Ideal(°)
1	C	319	ARG	NE-CZ-NH2	-8.08	116.26	120.30
1	C	288	ASP	CB-CG-OD2	-7.44	111.60	118.30
1	A	262	ARG	NE-CZ-NH1	6.86	123.73	120.30
1	C	262	ARG	NE-CZ-NH1	6.38	123.49	120.30
1	C	288	ASP	CB-CG-OD1	6.33	123.99	118.30
1	D	259[A]	ARG	NE-CZ-NH2	-6.24	117.18	120.30
1	D	259[B]	ARG	NE-CZ-NH2	-6.24	117.18	120.30
1	B	262	ARG	NE-CZ-NH1	6.12	123.36	120.30
1	C	317	MET	CG-SD-CE	6.06	109.90	100.20
1	B	319	ARG	NE-CZ-NH1	6.02	123.31	120.30
1	D	262	ARG	NE-CZ-NH1	6.01	123.30	120.30
1	A	322	MET	CG-SD-CE	-6.00	90.60	100.20
1	D	276	ARG	NE-CZ-NH1	5.90	123.25	120.30
1	D	259[A]	ARG	NE-CZ-NH1	5.89	123.25	120.30
1	D	259[B]	ARG	NE-CZ-NH1	5.89	123.25	120.30
1	A	317	MET	CG-SD-CE	5.83	109.53	100.20
1	B	242	LEU	CA-CB-CG	5.80	128.65	115.30
1	C	343	ASP	CB-CG-OD1	5.78	123.50	118.30
1	D	358	ASP	CB-CG-OD1	5.61	123.35	118.30
1	C	319	ARG	NE-CZ-NH1	5.58	123.09	120.30

Continued on next page...

CONFIDENTIAL VALIDATION REPORT

Continued from previous page...

Mol	Chain	Res	Type	Atoms	Z	Observed(°)	Ideal(°)
1	A	319	ARG	NE-CZ-NH2	-5.50	117.55	120.30
1	C	340	ASP	CB-CG-OD1	5.01	122.81	118.30

There are no chirality outliers.

There are no planarity outliers.

5.2 Too-close contacts (i)

In the following table, the Non-H and H(model) columns list the number of non-hydrogen atoms and hydrogen atoms in the chain respectively. The H(added) column lists the number of hydrogen atoms added and optimized by MolProbity. The Clashes column lists the number of clashes within the asymmetric unit, whereas Symm-Clashes lists symmetry-related clashes.

Mol	Chain	Non-H	H(model)	H(added)	Clashes	Symm-Clashes
1	A	904	0	887	1	0
1	B	982	0	965	4	0
1	C	893	0	875	11	0
1	D	893	0	877	5	0
2	C	32	0	12	0	0
3	A	101	0	0	1	0
3	B	126	0	0	2	0
3	C	106	0	0	1	0
3	D	109	0	0	2	0
All	All	4146	0	3616	17	0

The all-atom clashscore is defined as the number of clashes found per 1000 atoms (including hydrogen atoms). The all-atom clashscore for this structure is 2.

All (17) close contacts within the same asymmetric unit are listed below, sorted by their clash magnitude.

Atom-1	Atom-2	Interatomic distance (Å)	Clash overlap (Å)
1:C:334[B]:THR:HG22	1:D:334[B]:THR:HG23	1.13	1.06
1:C:334[B]:THR:CG2	1:D:334[B]:THR:HG23	2.04	0.84
1:C:334[A]:THR:OG1	1:D:334[A]:THR:HG22	1.83	0.77
1:C:288:ASP:H	1:C:356:HIS:HD2	1.29	0.76
1:C:334[A]:THR:HG23	3:D:465:HOH:O	1.92	0.70
1:B:242:LEU:N	3:B:401:HOH:O	2.25	0.70
1:C:345:ASN:ND2	3:C:501:HOH:O	2.30	0.64
1:C:319:ARG:HB2	1:C:334[A]:THR:HG22	1.83	0.60
1:D:259[B]:ARG:NH1	3:D:402:HOH:O	2.38	0.56

Continued on next page...

CONFIDENTIAL VALIDATION REPORT

Continued from previous page...

Atom-1	Atom-2	Interatomic distance (Å)	Clash overlap (Å)
1:C:360:TYR:HA	1:C:363:PHE:CZ	2.50	0.46
1:C:334[B]:THR:HG22	1:D:334[B]:THR:CG2	2.09	0.44
1:A:347:LYS:HG2	3:A:414:HOH:O	2.18	0.43
1:C:334[A]:THR:O	1:C:334[A]:THR:CG2	2.64	0.43
1:C:282:THR:O	1:C:282:THR:HG22	2.19	0.43
1:B:355:LYS:NZ	3:B:405:HOH:O	2.51	0.43
1:B:269:ASN:HB2	1:B:292:ILE:O	2.20	0.42
1:B:360:TYR:HA	1:B:363:PHE:CZ	2.56	0.40

There are no symmetry-related clashes.

5.3 Torsion angles (i)

5.3.1 Protein backbone (i)

In the following table, the Percentiles column shows the percent Ramachandran outliers of the chain as a percentile score with respect to all X-ray entries followed by that with respect to entries of similar resolution.

The Analysed column shows the number of residues for which the backbone conformation was analysed, and the total number of residues.

Mol	Chain	Analysed	Favoured	Allowed	Outliers	Percentiles	
1	A	111/136 (82%)	110 (99%)	1 (1%)	0	100	100
1	B	121/136 (89%)	119 (98%)	2 (2%)	0	100	100
1	C	110/136 (81%)	109 (99%)	1 (1%)	0	100	100
1	D	109/136 (80%)	107 (98%)	2 (2%)	0	100	100
All	All	451/544 (83%)	445 (99%)	6 (1%)	0	100	100

There are no Ramachandran outliers to report.

5.3.2 Protein sidechains (i)

In the following table, the Percentiles column shows the percent sidechain outliers of the chain as a percentile score with respect to all X-ray entries followed by that with respect to entries of similar resolution.

The Analysed column shows the number of residues for which the sidechain conformation was analysed, and the total number of residues.

CONFIDENTIAL VALIDATION REPORT

Mol	Chain	Analysed	Rotameric	Outliers	Percentiles	
1	A	94/113 (83%)	91 (97%)	3 (3%)	39	25
1	B	102/113 (90%)	101 (99%)	1 (1%)	76	71
1	C	93/113 (82%)	91 (98%)	2 (2%)	52	39
1	D	93/113 (82%)	88 (95%)	5 (5%)	22	9
All	All	382/452 (84%)	371 (97%)	11 (3%)	42	29

All (11) residues with a non-rotameric sidechain are listed below:

Mol	Chain	Res	Type
1	A	256	LYS
1	A	277	ARG
1	A	322	MET
1	B	277	ARG
1	C	256	LYS
1	C	277	ARG
1	D	256	LYS
1	D	277	ARG
1	D	280	GLU
1	D	322	MET
1	D	342	LYS

Sometimes sidechains can be flipped to improve hydrogen bonding and reduce clashes. All (5) such sidechains are listed below:

Mol	Chain	Res	Type
1	A	281	GLN
1	B	281	GLN
1	B	345	ASN
1	C	345	ASN
1	C	356	HIS

5.3.3 RNA ⓘ

There are no RNA molecules in this entry.

5.4 Non-standard residues in protein, DNA, RNA chains ⓘ

There are no non-standard protein/DNA/RNA residues in this entry.

CONFIDENTIAL VALIDATION REPORT

5.5 Carbohydrates ⓘ

There are no monosaccharides in this entry.

5.6 Ligand geometry ⓘ

1 ligand is modelled in this entry.

In the following table, the Counts columns list the number of bonds (or angles) for which Mogul statistics could be retrieved, the number of bonds (or angles) that are observed in the model and the number of bonds (or angles) that are defined in the Chemical Component Dictionary. The Link column lists molecule types, if any, to which the group is linked. The Z score for a bond length (or angle) is the number of standard deviations the observed value is removed from the expected value. A bond length (or angle) with $|Z| > 2$ is considered an outlier worth inspection. RMSZ is the root-mean-square of all Z scores of the bond lengths (or angles).

Mol	Type	Chain	Res	Link	Bond lengths			Bond angles		
					Counts	RMSZ	#$|Z|>2$	Counts	RMSZ	#$|Z|>2$
2	GTP	C	401	-	26,34,34	1.37	5 (19%)	33,54,54	2.18	8 (24%)

In the following table, the Chirals column lists the number of chiral outliers, the number of chiral centers analysed, the number of these observed in the model and the number defined in the Chemical Component Dictionary. Similar counts are reported in the Torsion and Rings columns. '-' means no outliers of that kind were identified.

Mol	Type	Chain	Res	Link	Chirals	Torsions	Rings
2	GTP	C	401	-	-	6/18/38/38	0/3/3/3

All (5) bond length outliers are listed below:

Mol	Chain	Res	Type	Atoms	Z	Observed(Å)	Ideal(Å)
2	C	401	GTP	C2-N2	3.15	1.40	1.33
2	C	401	GTP	C6-C5	2.88	1.46	1.41
2	C	401	GTP	C5-C4	2.59	1.47	1.40
2	C	401	GTP	O4'-C1'	2.50	1.44	1.41
2	C	401	GTP	C6-N1	2.42	1.37	1.33

All (8) bond angle outliers are listed below:

Mol	Chain	Res	Type	Atoms	Z	Observed(^{o})	Ideal(^{o})
2	C	401	GTP	N3-C2-N1	-5.43	119.98	127.22
2	C	401	GTP	C6-C5-C4	-5.32	115.72	120.80
2	C	401	GTP	C6-N1-C2	4.72	123.43	115.93
2	C	401	GTP	N2-C2-N3	4.31	124.82	117.79

Continued on next page...

CONFIDENTIAL VALIDATION REPORT

Continued from previous page...

Mol	Chain	Res	Type	Atoms	Z	Observed(^{o})	Ideal(^{o})
2	C	401	GTP	C2-N3-C4	4.30	120.27	115.36
2	C	401	GTP	PB-O3B-PG	-2.98	122.59	132.83
2	C	401	GTP	C5-C6-N1	-2.74	119.69	123.43
2	C	401	GTP	C3'-C2'-C1'	2.11	104.16	100.98

There are no chirality outliers.

All (6) torsion outliers are listed below:

Mol	Chain	Res	Type	Atoms
2	C	401	GTP	C5'-O5'-PA-O3A
2	C	401	GTP	C5'-O5'-PA-O1A
2	C	401	GTP	O4'-C4'-C5'-O5'
2	C	401	GTP	C3'-C4'-C5'-O5'
2	C	401	GTP	PG-O3B-PB-O1B
2	C	401	GTP	PB-O3A-PA-O2A

There are no ring outliers.

No monomer is involved in short contacts.

The following is a two-dimensional graphical depiction of Mogul quality analysis of bond lengths, bond angles, torsion angles, and ring geometry for all instances of the Ligand of Interest. In addition, ligands with molecular weight > 250 and outliers as shown on the validation Tables will also be included. For torsion angles, if less then 5% of the Mogul distribution of torsion angles is within 10 degrees of the torsion angle in question, then that torsion angle is considered an outlier. Any bond that is central to one or more torsion angles identified as an outlier by Mogul will be highlighted in the graph. For rings, the root-mean-square deviation (RMSD) between the ring in question and similar rings identified by Mogul is calculated over all ring torsion angles. If the average RMSD is greater than 60 degrees and the minimal RMSD between the ring in question and any Mogul-identified rings is also greater than 60 degrees, then that ring is considered an outlier. The outliers are highlighted in purple. The color gray indicates Mogul did not find sufficient equivalents in the CSD to analyse the geometry.

CONFIDENTIAL VALIDATION REPORT

Ligand GTP C 401

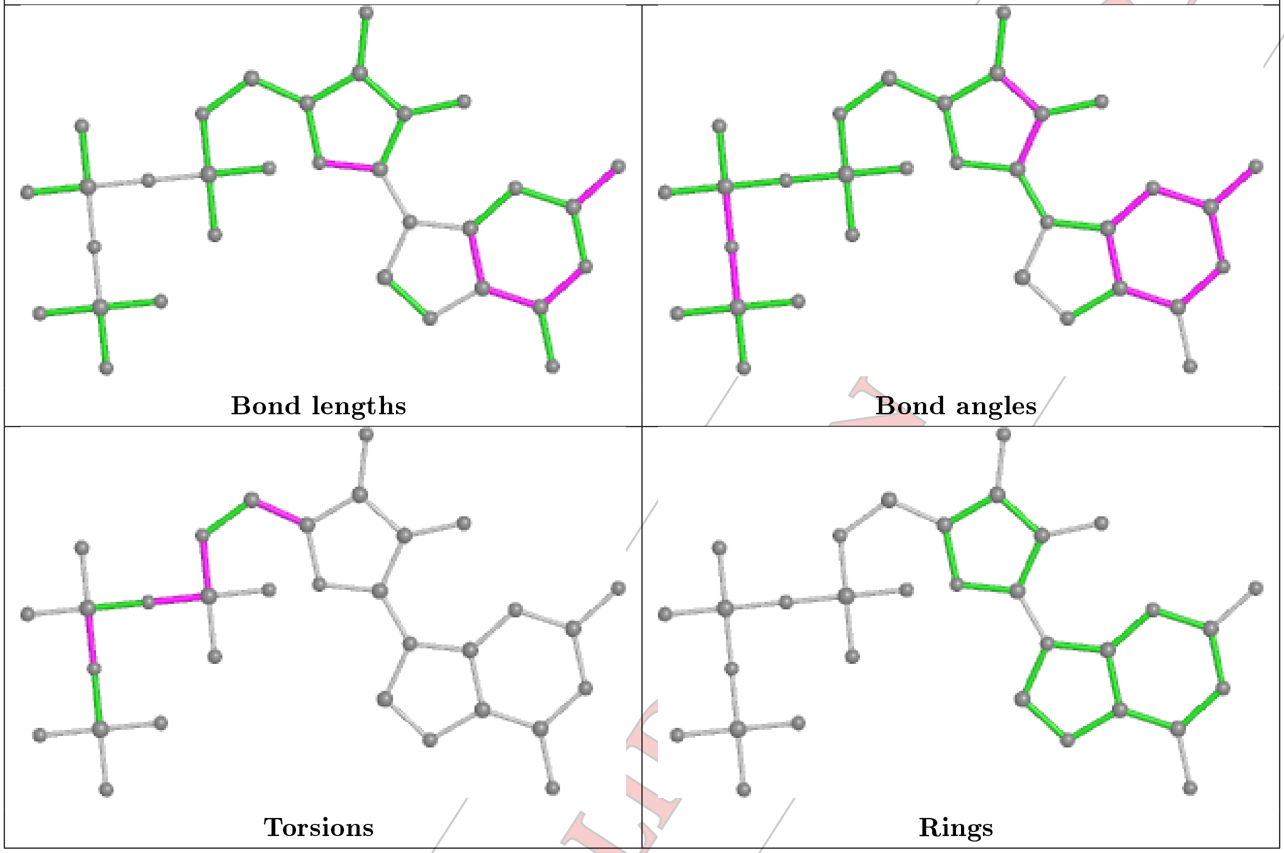

5.7 Other polymers (i)

There are no such residues in this entry.

5.8 Polymer linkage issues (i)

There are no chain breaks in this entry.

CONFIDENTIAL VALIDATION REPORT

6 Fit of model and data (i)

6.1 Protein, DNA and RNA chains (i)

In the following table, the column labelled '#RSRZ> 2' contains the number (and percentage) of RSRZ outliers, followed by percent RSRZ outliers for the chain as percentile scores relative to all X-ray entries and entries of similar resolution. The OWAB column contains the minimum, median, 95th percentile and maximum values of the occupancy-weighted average B-factor per residue. The column labelled 'Q< 0.9' lists the number of (and percentage) of residues with an average occupancy less than 0.9.

Mol	Chain	Analysed	<RSRZ>	#RSRZ>2			OWAB(Å^2)	Q<0.9
1	A	111/136 (81%)	-0.54	0	100	100	17, 26, 46, 75	0
1	B	123/136 (90%)	-0.46	0	100	100	16, 24, 44, 62	0
1	C	111/136 (81%)	-0.51	1 (0%)	84	82	17, 27, 44, 73	0
1	D	109/136 (80%)	-0.55	1 (0%)	84	82	18, 24, 43, 80	0
All	All	454/544 (83%)	-0.52	2 (0%)	92	90	16, 26, 47, 80	0

All (2) RSRZ outliers are listed below:

Mol	Chain	Res	Type	RSRZ
1	D	256	LYS	2.6
1	C	254	ALA	2.5

6.2 Non-standard residues in protein, DNA, RNA chains (i)

There are no non-standard protein/DNA/RNA residues in this entry.

6.3 Carbohydrates (i)

There are no monosaccharides in this entry.

6.4 Ligands (i)

In the following table, the Atoms column lists the number of modelled atoms in the group and the number defined in the chemical component dictionary. The B-factors column lists the minimum, median, 95th percentile and maximum values of B factors of atoms in the group. The column labelled 'Q< 0.9' lists the number of atoms with occupancy less than 0.9.

CONFIDENTIAL VALIDATION REPORT

Mol	Type	Chain	Res	Atoms	RSCC	RSR	B-factors($Å^2$)	Q<0.9
2	GTP	C	401	32/32	0.90	0.15	31,70,120,124	0

The following is a graphical depiction of the model fit to experimental electron density of all instances of the Ligand of Interest. In addition, ligands with molecular weight > 250 and outliers as shown on the geometry validation Tables will also be included. Each fit is shown from different orientation to approximate a three-dimensional view.

Electron density around GTP C 401:

$2mF_o$-DF_c (at 0.7 rmsd) in gray
mF_o-DF_c (at 3 rmsd) in purple (negative)
and green (positive)

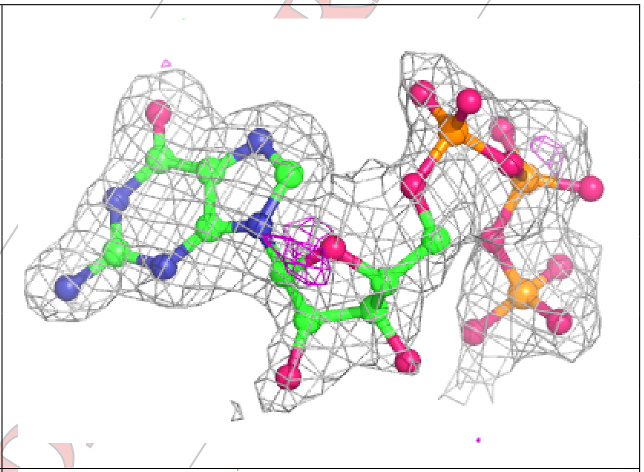

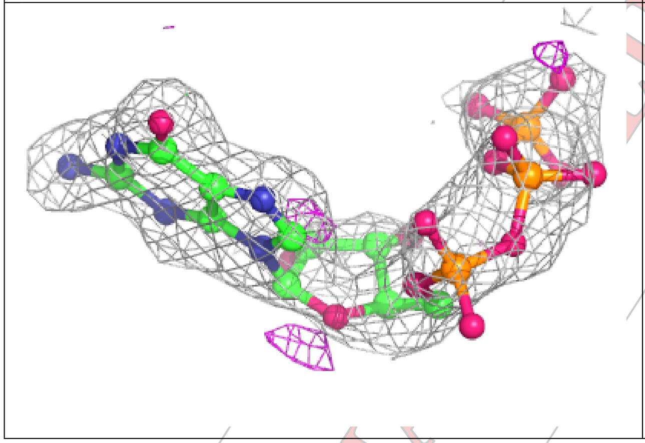

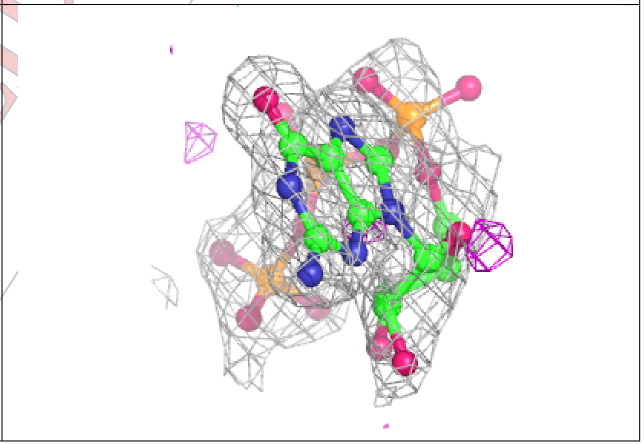

6.5 Other polymers ⓘ

There are no such residues in this entry.

CONFIDENTIAL REPORT
